# Myelin Oligodendrocyte Glycoprotein Antibody‐Associated Cerebral Cortical Encephalitis: A Comparative Study With Antibody‐Negative and Non‐MOG Antibody‐Positive Cortical Encephalitis in Chinese Adults

**DOI:** 10.1002/cns.70915

**Published:** 2026-05-06

**Authors:** Qing Yin, Binhong Han, Kai Su, Weiqi Dai, Jing Wu, Li Yang, Yuwei Dai, Dan Wang

**Affiliations:** ^1^ Department of Neurology, Second Xiangya Hospital Central South University Changsha Hunan China; ^2^ Clinical Medical Research Center for Stroke Prevention and Treatment of Hunan Province, Department of Neurology, Second Xiangya Hospital Central South University Changsha Hunan China; ^3^ Department of Neurology, Xiangya Hospital Central South University Changsha Hunan China; ^4^ Department of Radiology, Second Xiangya Hospital Central South University Changsha Hunan China; ^5^ National Center for Mental Disorders, National Clinical Research Center for Mental Disorders The Second Xiangya Hospital of Central South University Changsha China; ^6^ Department of Anesthesiology, Second Xiangya Hospital Central South University Changsha Hunan China

**Keywords:** autoimmune encephalitis, cerebral cortical encephalitis, MOGAD, myelin oligodendrocyte glycoprotein antibody, prognosis

## Abstract

**Aims:**

To investigate the clinical, radiological, and prognostic characteristics of autoimmune cortical encephalitis associated with different autoantibodies in Chinese adults, and to compare the features of myelin oligodendrocyte glycoprotein antibody–associated cerebral cortical encephalitis (MOG‐CCE) with antibody‐negative and non‐MOG antibody‐positive autoimmune cortical encephalitis.

**Methods:**

A retrospective study was conducted on 120 adult patients diagnosed with autoimmune cortical encephalitis at the Second Xiangya Hospital, Central South University, from 2014 to 2024. Patients were divided into three subgroups: the antibody‐negative group, the non‐MOG antibody‐positive group, and the MOG‐CCE group. Clinical, radiological, and therapeutic characteristics were analyzed, and logistic regression was performed to identify predictors of poor prognosis.

**Results:**

MOG‐CCE accounted for 18.3% of all autoimmune cortical encephalitis cases. Compared with other subtypes, MOG‐CCE was characterized by seizure‐dominant clinical presentation and unilateral cortical MRI lesions. Patients with MOG‐CCE responded favorably to immunotherapy and exhibited lower recurrence and disability rates. In contrast, antibody‐negative and non‐MOG antibody‐positive autoimmune cortical encephalitis showed more severe neuropsychiatric symptoms and poorer outcomes. A higher peak mRS score was the strongest independent predictor of long‐term disability.

**Conclusions:**

MOG‐CCE represents a distinct clinical phenotype within autoimmune cortical encephalitis, with favorable immunotherapy responsiveness and prognosis. Antibody profiling aids in disease classification and prognostic evaluation. Prospective multicenter studies are warranted to elucidate underlying mechanisms and refine individualized therapeutic strategies.

## Graphical abstract

MOG antibody‐associated cortical encephalitis (MOG‐CCE) presents a distinct clinical phenotype characterized by seizure‐dominant onset, unilateral cortical lesions, and favorable immunotherapy response, highlighting the diagnostic and prognostic value of antibody profiling in autoimmune cortical encephalitis.

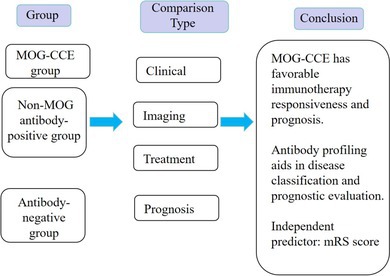

## 1 Introduction

Cortical encephalitis typically presents with fever, headache, seizures, and focal cortical deficits. Radiological findings are characterized by unilateral or bilateral cortical hyperintensities on T2‐weighted and fluid‐attenuated inversion recovery (T2‐FLAIR) sequences of brain magnetic resonance imaging (MRI) [1]. Autoimmune cortical encephalitis is frequently associated with autoantibodies directed against neuronal surface antigens, synaptic proteins, or intracellular epitopes, with variable responses to immunotherapy depending on the specific antibody profile [2].

With advances in antibody detection methodologies, more neuronal autoimmune antibodies have been identified in patients with autoimmune cortical encephalitis, including antibodies against N‐methyl‐D‐aspartate‐receptor (NMDAR) and leucine‐rich glioma‐inactivated 1(LGI‐1) [3]. Beyond these classical neuronal autoantibodies, cortical encephalitis has recently emerged as a novel phenotype of myelin oligodendrocyte glycoprotein antibody associated disease (MOGAD) [4, 5]. Since the initial report by Ogawa et al., several case series and case reports of MOG antibody‐associated cerebral cortical encephalitis (MOG‐CCE) have been published [4, 5, 6, 7]. However, studies focusing on adult cohorts with MOG‐CCE remain limited. Current understanding of this condition derives primarily from cohort studies exclusively focusing on MOG‐CCE with limited follow‐up periods [5, 6]. Comparative analyses of clinical characteristics, radiological patterns, treatment response, and long‐term outcomes of cortical encephalitis based on antibody status are scarce, particularly data from Chinese adult populations [8].

In the present study, cortical encephalitis was defined as a clinic‐radiological phenotype requiring both encephalitic symptoms and cortical signal abnormalities on MRI, either focal or diffuse, after careful exclusion of alternative etiologies. Included patients presented with acute or subacute encephalitic symptoms accompanied by diffuse or focal cortical hyperintensities on brain MRI and were subsequently stratified into subgroups based on antibody status. We aimed to delineate the clinical, radiological features, as well as outcomes of autoimmune cortical encephalitis associated with diverse autoantibodies in a Chinese tertiary medical center. Furthermore, we compared the distinctive characteristics of MOG‐CCE with antibody‐negative cortical encephalitis and non‐MOG antibody‐positive cortical encephalitis and explored risk factors predictive of poor outcomes in autoimmune cortical encephalitis. Our study was designed to characterize antibody‐associated clinical phenotypes rather than to establish direct causal relationships between specific antibodies and disease pathogenesis.

## 2 Method

### 2.1 Study Population

#### 2.1.1 Inclusion and Exclusion Criteria

A retrospective study was conducted at Second Xiangya Hospital of Central South University from January 2014 to June 2024. The study was approved by the Medical Ethics Committee of the Second Xiangya Hospital, Central South University, and was carried out in compliance with the Declaration of Helsinki. Written informed consents for inclusion in this study and publication of clinical details were obtained from all participants involved in this study.

Patients were included in this study if they met all of the following criteria: (1) age at disease onset ≥ 16 years; (2) acute or subacute presentation of encephalitic symptoms, including but not limited to altered consciousness, psychiatric manifestations, seizures, memory dysfunction, speech impairment, movement disorders, gait disturbance, or motor weakness; (3) testing for autoimmune antibodies had been performed (serum and/or cerebrospinal fluid (CSF)); (4) brain MRI was obtained during the acute phase of disease, defined as within 3 months of symptom onset [3]; and (5) presence of abnormal cortical hyperintensities on FLAIR or T2‐weighted MRI sequences.

Exclusion criteria were as follows: (1) presence of an alternative etiology for cortical encephalitis other than autoimmune causes, including infectious, metabolic/toxic, prion‐related, and paraneoplastic; (2) MRI examination performed beyond the acute phase (> 3 months after symptom onset); (3) absence of cortical hyperintensities on T2/FLAIR; (4) incomplete clinical documentation or inadequate follow‐up data.

#### 2.1.2 Diagnostic Work‐Up for Exclusion of Alternative Etiologies

All enrolled patients underwent a standardized and comprehensive diagnostic work‐up to exclude potential infectious, metabolic/toxic, prion‐related, and paraneoplastic causes of cortical encephalitis (Table S1).

For infectious screening, CSF samples were systematically analyzed using polymerase chain reaction (PCR) assays targeting common neurotropic viruses, including herpes simplex virus (HSV), varicella‐zoster virus (VZV), and human herpesvirus 6 (HHV‐6). In addition, metagenomic next‐generation sequencing (mNGS) of CSF was performed when clinically indicated. Conventional microbiological examinations of CSF included bacterial culture combined with Gram staining, fungal culture with India ink staining, and acid‐fast bacilli staining to assess possible mycobacterial infection.

To evaluate potential metabolic or toxic encephalopathies, serum levels of thiamine (vitamin B1) and vitamin B12 were measured in all patients. Thyroid function tests, with or without thyroid autoantibody profiling, were conducted to identify thyroid‐related encephalopathies.

In patients presenting rapidly progressive cognitive decline, CSF biomarkers were assessed when clinically indicated to support the differential diagnosis of prion disease. Specifically, CSF 14‐3‐3 protein and total tau levels were measured, and real‐time quaking‐induced conversion (RT‐QuIC) assays were performed when available to further improve diagnostic accuracy and specificity for prion disease.

To exclude paraneoplastic etiologies, all patients underwent systematic tumor screening, including testing serum and/or CSF samples for classical paraneoplastic antibodies, including anti‐Hu, anti‐Yo, anti‐Ri, anti‐Ma2, and anti‐CV2/CRMP5 antibodies. All aforementioned investigations were completed, and no alternative etiology was identified in the enrolled patients.

#### 2.1.3 Grouping

According to the antibody status, the final research subjects were further categorized into three groups based on antibody status and existing diagnostic criteria: the antibody‐negative group (*n* = 49, 40.8%) [9, 10], the non‐MOG antibody‐positive group (*n* = 49, 40.8%) [11], and the MOG‐CCE group (*n* = 22, 18.3%) [12]. Additionally, patients were stratified into two subgroups based on their Modified Rankin Scale (mRS) score at the last follow‐up: patients with favorable long‐term outcome (mRS < 3.0); patients with poor long‐term outcome (mRS ≥ 3.0) [13].

### 2.2 Clinical Data Collection

All data were retrospectively extracted from electronic medical records and systematically organized into standardized data collection forms. The following information was collected: (1) Demographic characteristics: Gender and age at disease onset. (2) Clinical presentations: (A) Duration from disease onset to symptom peak; (B) Encephalitic manifestations, including psychiatric symptoms, seizures, altered consciousness, movement disorders (dyskinesia/dystonia), speech dysfunction, memory impairment, autonomic dysfunction, and gait disturbances/ataxia; (C) Clinical severity assessments: Clinical symptom severity was evaluated using the Clinical Assessment Scale in Autoimmune Encephalitis (CASE, score range 0–27) [13, 14], and neurological functional disability was assessed using mRS (score range 0–6) [15]. Both assessments were performed retrospectively by two independent neurologists (Qing Yin and Yuwei Dai) at disease peak and final follow‐up; (D) Presence of tumors; (E) Critical care requirements, including intensive care unit (ICU) admission and mechanical ventilation. (3) Paraclinical investigations: CSF analysis, autoimmune antibody profiles, and MRI patterns. CSF abnormalities were defined as a white blood cell count > 5 cells/μL, elevated CSF protein concentration (> 450 mg/L), or increased total cell count, with negative CSF bacterial and fungal cultures. None of the above conditions is considered normal CSF findings [16]. (4) Clinical outcomes: Outcome was determined by mRS score at the last follow‐up, with poor prognosis defined as mRS ≥ 3. (5) Therapeutic interventions: Therapeutic interventions were categorized into acute phase treatment and maintenance immunotherapy according to the International MOGAD Consensus [17]. Acute phase treatment, targeting acute neurological attacks and accelerating functional recovery, comprised high‐dose intravenous methylprednisolone (IVMP), intravenous immunoglobulin (IVIg), and plasma exchange (PLEX). Maintenance immunotherapy, aimed at long‐term relapse prevention, included mycophenolate mofetil (MMF), azathioprine (AZA), and cyclophosphamide (CYC) administered orally, as well as intravenous rituximab (RTX).

### 2.3 Review of Brain MRI

Brain MRIs were obtained using a 3.0‐Tesla or 1.5‐Tesla MAGNETOM Verio scanner (Siemens, Germany) at the Second Xiangya Hospital of Central South University. The following sequences were acquired: T1‐weighted images (T1WI), T2WI, FLAIR, diffusion‐weighted images (DWI), and gadolinium‐enhanced images. Lesions were primarily observed in the cortex and medial temporal regions, and lesion locations were recorded. MRIs were independently reviewed in a blinded manner by two trained neuroradiologists.

### 2.4 Antibody Testing

All patients underwent testing for MOG antibodies in both serum and cerebrospinal fluid, as well as an autoimmune encephalitis‐related panel. The panel included neuronal cell‐surface antibodies (NMDAR, LGI‐1, contactin‐associated protein‐2 [CASPR2], α‐amino‐3‐hydroxy‐5‐methyl‐4‐isoxazole propionic acid receptor [AMPAR], γ‐aminobutyric acid type A receptor [GABAAR], γ‐aminobutyric acid type B receptor [GABABR], dipeptidyl‐peptidase‐like protein‐6 [DPPX], glycine receptor [GlyR]), IgLON family member 5 [IgLON5], and intracellular antibodies (such as glutamic acid decarboxylase 65‐kDa isoform [GAD65], SOX1, Hu, Yo, Ri, Ma1, Ma2, CV2, amphiphysin). Antibody testing was performed at Guangzhou Jinyu Medical Laboratory Center.

Autoimmune encephalitis‐related antibodies in serum and cerebrospinal fluid were analyzed using cell‐based assays (CBA). Cell‐surface antibodies were detected by indirect immunofluorescence, whereas intracellular antibodies were assessed by immunoblotting. For MOG‐IgG testing, antibody levels were determined using a live CBA with serial serum and/or CSF dilutions. Titers were expressed as end‐point dilutions. In accordance with commonly used criteria in recent MOG‐IgG–associated disease studies [18, 19], results were categorized as follows: negative, defined as no specific immunofluorescence signal at a dilution of 1:10; low‐positive, defined as detectable positivity at dilutions of 1:10 to < 1:100; and high‐positive, defined as positivity at dilutions ≥ 1:100. This stratification was applied to facilitate standardized interpretation of MOG‐IgG results and subsequent subgroup analyses. Quantitative antibody titers for all antibody‐positive cases, including median values and ranges, are summarized in Table S2. All stained slides were independently reviewed by two experienced neuroimmunologists. Samples positive for any antibody underwent at least two independent confirmatory tests performed at different time points or by different laboratory personnel. Antibody‐negative was defined as the absence of well‐characterized neuronal autoantibodies in serum or cerebrospinal fluid as determined by validated cell‐based assays. All samples were collected after admission and prior to initiation of immunotherapy.

### 2.5 Statistical Analysis

Quantitative variables were expressed as mean ± standard deviation or median (interquartile range), as appropriate. Categorical variables were summarized as frequencies and percentages. For statistical analyses, categorical variables with more than two categories were recoded into dummy variables for both group comparisons and regression analyses.

For intergroup comparisons, the chi‐square test or Fisher's exact test was used for categorical variables, while nonparametric tests (Kruskal–Wallis or Wilcoxon rank‐sum test) were applied for continuous variables depending on data distribution. To adjust for multiple comparisons, the Benjamini–Hochberg procedure was employed to control the false discovery rate (FDR). If overall differences were significant, post hoc pairwise comparisons were conducted to explore the distinction of the MOG‐antibody‐positive group using Bonferroni correction. Both FDR and Bonferroni adjustments were performed in R software.

Univariate binary logistic regression was conducted to identify potential risk factors for poor prognosis. Variables with *p* ≤ 0.05 in univariate analysis were further screened using least absolute shrinkage and selection operator (LASSO) regression with the glmnet package in R. Variables retained by LASSO were subsequently entered into the multivariate logistic regression model. Results were reported as odds ratios (ORs) with 95% confidence intervals (CIs). All statistical analyses were performed using SPSS (version 26.0) and R (version 4.3.0). A two‐sided *p* < 0.05, FDR < 0.05, or Bonferroni‐corrected *p* < 0.017 was considered statistically significant.

## 3 Results

### 3.1 Participants Enrollment Flow

**FIGURE 1:**
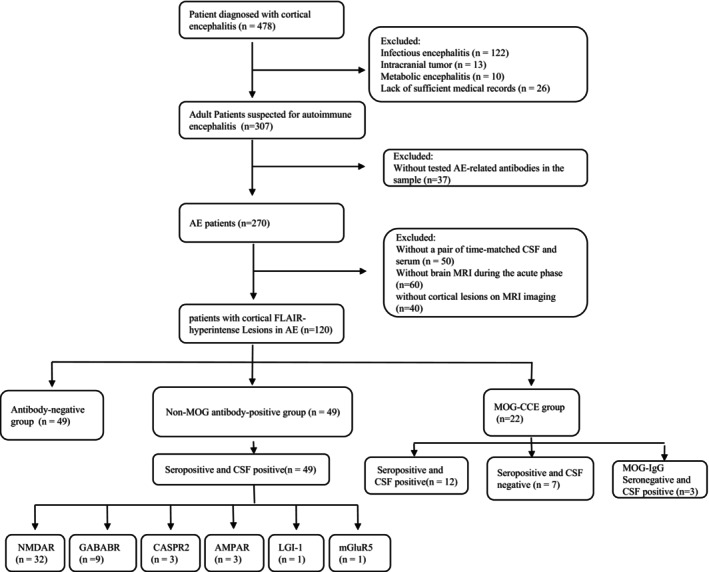
Flowchart of enrolled patients in our study.

A total of 478 adult patients with cortical encephalitis were initially enrolled in this study. Sequential exclusion criteria were applied as follows: First, 145 patients were excluded based on etiological analysis, comprising 122 patients diagnosed with infectious encephalitis, 13 with intracranial tumors, and 10 with cerebrovascular disease. Twenty‐six cases were excluded because of incomplete medical records. Among the remaining 307 patients with suspected autoimmune encephalitis (AE), 187 patients were further excluded: 37 patients who refused AE‐related autoantibody testing, 50 patients without a pair of time‐matched CSF and serum samples, 60 patients without brain MRI during the acute phase, and 40 patients with no cortical lesions. The final study cohort consisted of 120 patients with autoimmune cortical encephalitis (Figure 1).

### 3.2 Demographic Information and Clinical Characteristics

In our cohort, 63/120 (52.5%) were male. The mean age was 34.74 ± 15.47 years (range, 16–75). Sixteen patients (13.3%) were admitted to ICU, and 11 patients (9.1%) required mechanical ventilation.

In acute stage of autoimmune cortical encephalitis, the main symptoms of this cohort were seizures (62/120, 51.7%) and psychiatric symptoms (56/120, 46.7%), followed by impaired consciousness (51/120, 42.5%), dystonia (46/120, 38.3%), speech dysfunction (41/120, 34.2%), memory deficit (32/120, 26.7%), brainstem dysfunction (27/120, 27.5%) and ataxia (23/120, 19.2%). Neoplasms were found in 27 patients (22.5%).

### 3.3 CSF Profile and MRI Patterns

All patients underwent lumbar puncture during hospitalization. Among these, 40 patients (33.3%) had normal CSF findings. The median CSF protein level was 0.46 g/dL (IQR 0.21–0.58), and 64 patients (53.5%) had CSF pleocytosis (≥ 5 cells/μL).

MRIs were performed at a median 12 days (IQR 7–20) after disease onset. Fifty‐one patients (42.5%) showed bilateral cortical involvement, and 69 patients (57.5%) had unilateral cortical involvement. Abnormal T2 FLAIR hyperintensities were most frequently observed in the temporal lobe (*n* = 71, 59.2%), followed by the frontal lobe (*n* = 60, 50%), parietal lobe (*n* = 48, 40%), and occipital lobe (*n* = 19, 15.8%).

### 3.4 Antibody Detection

**FIGURE 2:**
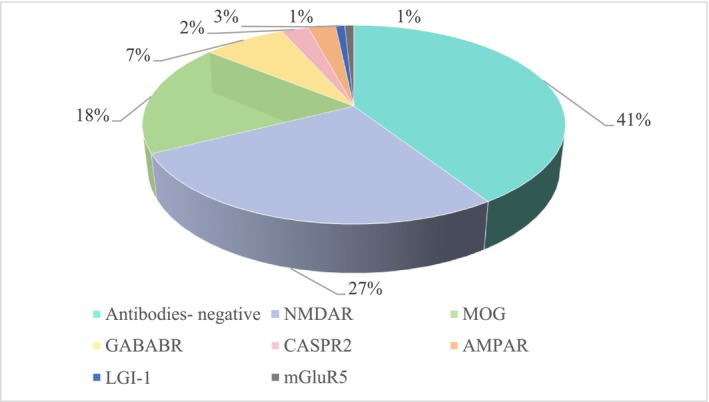
Pie chart. Neural antibody frequency among patients with autoimmune cortical encephalitis.

All patients underwent antibody detection both in CSF and serum during the acute phase (120/120,100%). Forty‐nine patients (40.8%) were the antibody‐negative group, 22 patients (18.3%) were the MOG‐CCE group, and 49 patients (59.2%) were the non‐MOG antibody‐positive group (Table 1). In the MOG‐CCE group, 12 patients (10%) were positive for MOG‐IgG paired serum and CSF positive, 7 patients (5.8%) were MOG‐IgG seropositive and CSF negative, and 3 patients (2.5%) were in CSF‐restricted MOG‐IgG (Figure 1). Forty‐nine patients with positive autoantibodies other than MOG antibodies were double positive for CSF and serum: NMDAR (*n* = 32), GABABR (*n* = 9), CASPR2 (*n* = 3), AMPAR (*n* = 3), LGI‐1 (*n* = 1), and mGluR5 (*n* = 1) (Figure 2). The included patients did not exhibit dual or multiple antibody positivity.

**TABLE 1:** Demographic and clinical data of patients with autoimmune cortical encephalitis.

Patients with autoimmune cortical encephalitis (*n* = 120)	
**Demographic information**	
Age at onset (years), medin ± SD	34.74 ± 15.47
M:F ratio	63: 57
ICU admission rate, *n* (%)	16 (13.3%)
Mechanical ventilation, *n* (%)	11 (9.1%)
**Clinical data**	
Time from onset to symptoms nadir (m), median[IQR]	0.75 [0.46–3]
Psychiatric symptom, *n* (%)	56 (46.7%)
Seizure, *n* (%)	62 (51.7%)
Impaired consciousness, *n* (%)	51 (42.5%)
Dyskinesia/dystonia, *n* (%)	46 (38.3%)
Speech dysfunction, *n* (%)	41 (34.2%)
Memory disturbance, *n* (%)	32 (26.7%)
Autonomic Symptoms, *n* (%)	27 (22.5%)
Gait instability/ataxia, *n* (%)	23 (19.2%)
Neoplasms, *n* (%)	27 (22.5%)
**Brain MRI findings**	
Time from onset to MRI (day), median [IQR]	12 [7–20]
Bilateral cortical involvement, *n* (%)	51 (42.5%)
Unilateral cortical involvement, *n* (%)	69 (57.5%)
**Lesion location**	
Temporal lobe, *n* (%)	71 (59.2%)
Frontal lobe, *n* (%)	60 (50%)
Parietal lobe, *n* (%)	48 (40%)
Occipital lobe, *n* (%)	19 (15.8%)
**CSF data**	
Time from onset to sampling of CSF (day), median [IQR]	12 [7–20]
Normal CSF, *n* (%)	40 (33.3%)
Pleocytosis, *n* (%)	64 (53.3%)
Protein (g/dL), median(range)	0.46 [0.03–2.27]
**Antibody status**	
Antibodies—negative, *n* (%)	49 (40.8%)
MOG‐antibody‐positive, *n* (%)	22 (18.3%)
Positive autoantibodies other than MOG antibodies, *n* (%)	49 (40.8%)
NMDAR, *n* (%)	32 (26.7%)
GABABR, *n* (%)	9 (7.5%)
CASPR2, *n* (%)	3 (2.5%)
AMPAR, *n* (%)	3 (2.5%)
LGI‐1, *n* (%)	1 (0.83%)
mGluR5, *n* (%)	1 (0.83%)
**Disease course and outcome**	
Follow‐up (mo), median [IQR]	11.68 [10–13]
Time from onset to treatment (day), median [IQR]	20 [12.45–27]
Relapse, *n* (%)	42 (35%)
**CASE and mRS scores**	
Peak CASE scores, median [IQR]	5 [3–8]
Peak mRS scores, median [IQR]	4 [3–4]
CASE score at last follow‐up, median [IQR]	2 [1–4]
mRS score at last follow‐up, median [IQR]	2 [1–3]
mRS scores ≥ 3 at the last follow‐up, *n* (%)	55 (45.8%)

Abbreviations: CASE, Clinical Assessment Scale in Autoimmune Encephalitis; CSF, cerebrospinal fluid; ICU, intensive care unit; mRS, modified Rankin Scale.

### 3.5 Changes in CASE and mRS

The median time from symptom onset to the nadir was 12.5 days (range 1–29 days), at which point the CASE score was 5 (IQR, 3–8) and mRS score was 4 (IQR, 3–4). After the median follow‐up time of 11.68 months (IQR: 10–13) since disease onset, the median mRS score was 2 (IQR, 1–3) and CASE score was 2 (IQR, 1–4).

**FIGURE 3:**
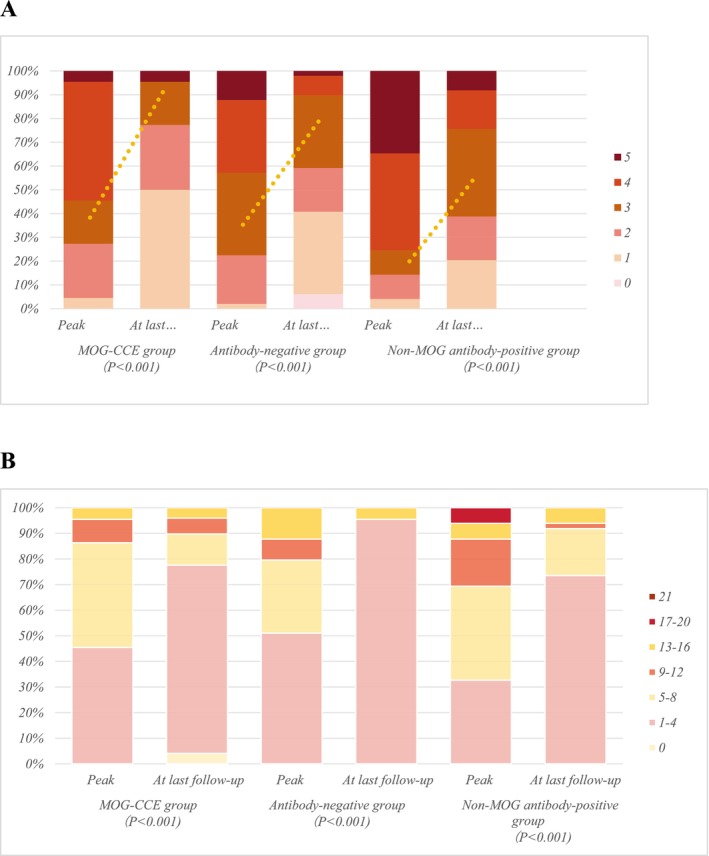
The mRS and CASE scores of three groups (MOG‐CCE; non‐MOG antibody‐positive; antibodies‐negative) at two time points, namely, at peak and at the last follow‐up. (A) The mRS score profiles of three groups at two time points; the dotted line indicates the trend in change for mRS scores ≥ 3 at peak and at the last follow‐up. (B) The CASE score profiles at three time points.

In subgroup analysis, the median CASE score at peak was 5 (IQR, 2–7.25) in the MOG‐CCE group, 6 (IQR, 3–9) in the non‐MOG antibody‐positive group, and 4 (IQR, 3–7) in the antibody‐negative group. At last follow‐up, the corresponding median CASE scores were 1 (IQR, 1–3), 2 (IQR, 2–5), and 2 (IQR, 1–4), respectively. For mRS scores, the median score at peak was 4 (IQR, 2–4) in the MOG‐CCE group, 4 (IQR, 3.5–5) in the non‐MOG antibody‐positive group, and 3 (IQR, 3–4) in the antibody‐negative group. At last follow‐up, the corresponding median mRS scores were 1.5 (IQR, 1–2.25), 3 (IQR, 2–3.5), and 2 (IQR, 1–3), respectively. The proportion of patients with mRS scores ≥ 3 decreased from peak to last follow‐up across all three groups. The largest reduction was observed in the antibody‐negative group (31.67% vs. 16.6%, *p* < 0.001), followed by the non‐MOG antibody‐positive group (35.01% vs. 25%, *p* < 0.001). The least significant decrease was found in the MOG‐CCE group (13.33% vs. 4.16%, *p* < 0.001). The change and distribution of mRS score and CASE score in three groups are displayed in Figure 3.

### 3.6 Comparison of Demographic Information and Clinical Data Among the Antibody‐Negative Group, Non‐MOG Antibody‐Positive Group, and the MOG‐CCE Group

Table 2 summarizes the comparison of demographic information and clinical characteristics of the three subgroups. The mean age of onset was comparable among the MOG‐CCE group (30 ± 13.75 years), the non‐MOG antibody‐positive group (30 ± 16.4 years), and the antibody‐negative group (34 ± 15.03 years) (*p* = 0.77; FDR‐*p* = 0.80). No significant differences were observed among the three groups regarding sex ratio, ICU admission rates, or the need for mechanical ventilation.

**TABLE 2:** Comparison of demographic and clinical data to different antibody states.

	MOG‐CCE group	Non‐MOG antibody‐positive group	Antibody‐negative group	*p*	FDR‐*p*
	*n* = 22	*n* = 49	*n* = 49		
Age at onset (years), mean ± SD	30 ± 13.75	30 ± 16.40	34 ± 15.03	0.77	0.80
Male:Female ratio	15:7	24:25	24:25	0.27	0.35
ICU admission rate, *n* (%)	2/22	7/49	7/49	0.81	0.81
Mechanical ventilation, *n* (%)	1/22	4/49	6/49	0.55	0.68
Peak CASE score, median (range)	5[1–14]	6 [1–18]	4[1–14]	0.10	0.16
Peak mRS score, median (range)	4[1–5]^a^, ^c^	4[1–5]	3[1–5]	**0.003** *	**0.01** *
Psychiatric symptom, *n* (%)	6/22^b^	33/49	17/49	**< 0.001** *	**0.01** *
Seizure, *n* (%)	11/22^b^	36/49	15/49	**< 0.001** *	**0.01** *
Impaired consciousness, *n* (%)	5/22^b^	18/49	28/49	**0.01** *	**0.03** *
Dyskinesia/dystonia, *n* (%)	11/22^b^	29/49	6/49	**< 0.001** *	**0.01** *
Speech dysfunction, *n* (%)	3/22	22/49	16/49	**0.03** *	0.06
Memory disturbance, *n* (%)	2/22	12/49	18/49	**0.03** *	0.06
Autonomic symptoms, *n* (%)	5/22	5/49	17/49	**0.02** *	0.05
Gait instability/ataxia, *n* (%)	5/22	16/49	2/49	**< 0.001** *	**0.01** *
Neoplasms, *n* (%)	10/22	9/49	13/49	0.06	0.10
Bilateral cortical involvement, *n* (%)	7/22^a^, ^b^	19/49	25/49	**< 0.001** *	**0.01** *
Unilateral cortical involvement, *n* (%)	15/22^a^, ^b^	30/49	24/49	**< 0.001** *	**0.01** *
Temporal lobe, *n* (%)	16/22	24/49	31/49	0.13	0.20
Frontal lobe, *n* (%)	10/22	27/49	23/49	0.65	0.70
Parietal lobe, *n* (%)	9/22	15/49	24/49	0.18	0.26
Occipital lobe, *n* (%)	3/22	5/49	11/49	0.24	0.33
Normal CSF, *n* (%)	3/22	14/49	23/49	**0.02** *	0.05
Pleocytosis, *n* (%)	15/22	19/49	30/49	**0.03** *	0.06
Protein concentration, mean (range)	0.49 [0.23–1.34]	0.35 [0.032–2.27]	0.29 [0.034–1.64]	**0.02** *	0.05

*Note:* Bold values have the same meaning as * (*p* < 0.05; FDR‐*p* < 0.05).

Abbreviations: CASE, Clinical Assessment Scale in Autoimmune Encephalitis; CSF, cerebrospinal fluid; ICU, intensive care unit; MOG‐CCE, myelin oligodendrocyte glycoprotein antibody‐associated cerebral cortical encephalitis; mRS, modified Rankin Scale.

^a^ The difference appears between the non‐MOG antibody‐positive group and MOG‐CCE group.

^b^ The difference appears between the MOG‐CCE group and Antibody‐negative group.

^c^ Cohen's *d* = 0.60.

* *p* < 0.05 FDR‐*p* < 0.05.

There was a significant overall difference in peak mRS scores among three groups (*p* = 0.003; FDR‐*p* = 0.01). Compared with the MOG‐CCE group, peak CASE scores were lower in the antibody‐negative group (*p* = 0.01). In contrast, scores in the non‐MOG antibody‐positive group did not differ from those in the MOG‐CCE group (*p* = 0.938).

At disease onset, compared with the MOG‐CCE group, the antibody‐negative group had significantly higher incidences of psychiatric symptoms (*p* = 0.002), and impaired consciousness (*p* = 0.007), but a significantly lower incidence of seizures (*p* < 0.001) and dyskinesia/dystonia (*p* < 0.001). Similarly, compared with the MOG‐CCE group, patients with positive autoantibodies other than MOG antibodies showed higher incidences of psychiatric symptoms, seizures, impaired consciousness, and dyskinesia/dystonia, although these differences were not statistically significant. No significant differences were observed among the groups in the incidences of speech dysfunction (*p* = 0.03; FDR‐*p* = 0.06), memory disturbance (*p* = 0.03; FDR‐*p* = 0.06), or autonomic symptoms (*p* = 0.02; FDR‐*p* = 0.05), nor in the prevalence of concurrent tumors (*p* = 0.06; FDR‐*p* = 0.10).

CSF analyses revealed no significant differences in protein concentration, pleocytosis, or the proportion of patients with normal CSF across the three groups (all FDR‐*p* > 0.05). Regarding brain MRI, compared with the MOG‐CCE group, both the antibody‐negative group (*p* < 0.001) and the non‐MOG antibody‐positive group (*p* < 0.001) showed a higher frequency of bilateral lesions and a lower frequency of unilateral involvement. No significant differences were observed in the distribution of lesions across specific brain lobes.

### 3.7 Comparison of Outcomes and Treatments Among the Antibody‐Negative Group, the Non‐MOG Antibody‐Positive Group, and the MOG‐CCE Group

Table 3 summarizes the outcomes and treatment comparisons across the three antibody groups. The median follow‐up time for all patients was 11.68 months (IQR 10–13), during which 42 patients (35%) experienced disease recurrence. It differed significantly among the three groups (*p* < 0.001; FDR‐*p* = 0.003). The recurrence rate in the MOG‐CCE group (5/22, 22.7%) was lower than that in the antibody‐negative group (20/49, 40.8%) and the non‐MOG antibody‐positive group (17/49, 34.7%).

**TABLE 3:** Outcome and treatment comparison of different antibody states.

	MOG‐CCE group	Non‐MOG antibody‐positive group	Antibody‐negative group	*p*	FDR‐*p*
	*n* = 22	*n* = 49	*n* = 49		
Follow‐up (mo) median, (range)	12 [6–24]	12 [6–24]	12 [6–23]	0.24	0.27
Relapse, *n* (%)	5/22^a^, ^b^	17/49	20/49	**< 0.001** *	**0.003** *
CASE at the last follow‐up, median, (range)	1 [1–13]^a^, ^c^	2 [1–15]	2 [0–14]	**0.01** *	**0.02** *
mRS at the last follow‐up, median, (range)	1.5 [1–5]^a^, ^d^	3 [1–5]	2 [0–5]	**0.003** *	**0.01** *
mRS ≥ 3.0 at the last follow up, *n* (%)	5/22^b^	20/49	30/49	**0.01** *	**0.02** *
Time from onset to treatment (day) median, (range)	21 [9–31]	20 [3–30]	16 [2–30]	0.2	0.26
Not treated	1/22 (4.5%)	3/49 (6.1%)	1/49 (2.0%)	0.77	0.77
Acute phase treatment only, *n* (%)	14/22^a^, ^b^ (63.6%)	30/49 (61.2%)	39/49 (79.6%)	**< 0.001** *	**0.003** *
IVMP	4/22 (18.2%)	19/49 (38.8%)	14/49 (28.6%)		
IVIg	14/22 (63.6%)	30/49 (61.2%)	39/49 (79.6%)		
Plasma exchange	1/22 (4.5%)	5/49 (10.2%)	7/49 (14.3%)		
Combined with maintenance immunotherapy, *n* (%)	7/22^a^, ^b^ (31.8%)	16/49 (32.7%)	9/49 (18.4%)	**< 0.001** *	**0.003** *
Azathioprine	6/22 (27.3%)	7/49 (14.3%)	3/49 (6.12%)		
Mycophenolate mofetil	6/22 (27.3%)	7/49 (14.3%)	3/49 (6.12%)		
Rituximab	3/22 (13.6%)	4/49 (8.16)	2/49 (4.08%)		
Cyclophosphamide	0	2/49 (4.08%)	1/49 (2.04%)		

*Note:* Bold values have the same meaning as * (*p* < 0.05; FDR‐*p* < 0.05).

Abbreviations: CASE, Clinical Assessment Scale in Autoimmune Encephalitis; CSF, cerebrospinal fluid; IVIg, intravenous immunoglobulin; IVMP, intravenous methylprednisolone; MOG‐CCE, myelin oligodendrocyte glycoprotein antibody‐associated cerebral cortical encephalitis; mRs, Modified Rankin Scale.

^a^ The difference appears between the non‐MOG antibody‐positive group and MOG‐CCE group.

^b^ The difference appears between the MOG‐CCE group and Antibodies—negative group.

^c^ Cohen's *d* = 0.55.

^d^ Cohen's *d* = 0.21.

* *p* < 0.05. FDR‐*p* < 0.05.

The mRS scores at the last follow‐up differed significantly among the three groups overall (*p* = 0.003; FDR‐*p* = 0.01). Compared with the MOG‐CCE group, the peak CASE score in the non‐MOG antibody‐positive group was significantly higher (*p* = 0.005), whereas no significant differences were observed in the other pairwise comparisons.

Significant differences were also observed in treatment strategies, specifically in the use of acute phase treatment alone (*p* < 0.001; FDR‐*p* = 0.003) and combined acute phase treatment and maintenance immunotherapy (*p* < 0.001; FDR‐*p* = 0.003). Compared with the MOG‐CCE group, the proportion of patients receiving acute phase treatment alone was significantly lower in the antibody‐negative group (63.6% vs. 61.2%, *p* < 0.001) and in the non‐MOG antibody‐positive group (63.6% vs. 79.6%, *p* < 0.001). In addition, the proportion of patients receiving combination therapy was significantly lower in the MOG‐CCE group than in the non‐MOG antibody‐positive group (31.8% vs. 32.7%, *p* < 0.001) and the antibody‐negative group (31.8% vs. 18.4%, *p* < 0.001). The proportion of untreated patients in the MOG‐CCE group (1/22, 4.54%) was lower than the non‐MOG antibody‐positive group (3/49, 6.12%) but higher than that in the antibody‐negative group (1/49, 2.04%); however, these differences were not statistically significant (*p* = 0.77; FDR‐*p* = 0.77).

### 3.8 Independent Risk Factors to Predict Poor Prognosis at the Last Follow‐Up

Logistic regression analysis was performed to identify risk factors associated with poor prognosis. Univariate analysis in Table 4 showed that admission to the intensive care unit (OR 4.26; 95% CI 1.29–14.09), higher peak CASE scores (OR 1.41; 95% CI 1.21–1.64), higher peak mRS scores (OR 4.61; 95% CI 1.69–7.97), more severe seizures (OR 3.14; 95% CI 1.49–6.65), autonomic symptoms (OR 3.03; 95% CI 1.23–7.46), impaired consciousness (OR 2.51; 95% CI 1.20–5.29), psychiatric symptoms (OR 2.51; 95% CI 1.19–5.09), and time from onset to treatment (OR 1.29; 95% CI 1.18–1.14) were significantly associated with an increased risk of poor outcomes (mRS ≥ 3) at the last follow‐up. Compared with the MOG‐CCE group, the non‐MOG antibody‐positive group (OR 2.91; 95% CI 1.37–6.17) was also significantly associated with adverse outcomes.

**TABLE 4:** Univariate logistic regression analysis for reaching mRS ≥ 3 at the last follow‐up.

Univariate analysis			
	OR	95% CI	*p*
Age (years)	0.99	0.97–1.02	0.67
Male sex	1.02	0.50–2.09	0.96
Admission to ICU	4.26	1.29–14.09	**0.02** *
Follow‐up (mo) median, (range)	0.98	0.90–1.08	0.69
Peak CASE scores	1.41	1.21–1.64	**< 0.01** *
Peak mRS scores	4.61	2.69–7.97	**< 0.01** *
Seizure	3.14	1.49–6.65	**0.003** *
Autonomic Symptoms	3.03	1.23–7.46	**0.02** *
Psychiatric symptoms	2.51	1.20–5.29	**0.02** *
Impaired consciousness	2.51	1.19–5.09	**0.02** *
Bilateral Cortical Involvement	1.30	0.63–2.67	0.47
Normal CSF	1.11	0.52–2.37	0.80
**Antibodies status**			
Antibodies‐ negative vs. MOG‐antibody‐positive	1.41	0.68–2.94	0.36
Non‐MOG antibodies vs. MOG‐antibody‐positive	2.91	1.37–6.17	**0.006** *
**Treatment**			
Time from onset to treatment (day) median	1.29	1.18–1.41	**< 0.01** *
Only first‐line immunotherapy vs. not treated	1.30	0.62–2.75	0.486
Combined with second‐line immunotherapy vs. Not treated	1.38	0.64–2.98	0.407

*Note:* Bold values have the same meaning as *(*p* < 0.05).

Abbreviations: CASE, Clinical Assessment Scale in Autoimmune Encephalitis; CSF, cerebrospinal fluid; ICU, intensive care unit; MOG, myelin oligodendrocyte glycoprotein; mRS, modified Rankin Scale.

* *p* < 0.05.

LASSO regression further selected three variables (peak CASE score, peak mRS score, and antibody status), which were included in the multivariate binary logistic regression model. The results demonstrated that higher peak mRS score was the strongest independent predictor of poor prognosis (OR 3.14; 95% CI 1.59–6.20; *p* = 0.001). In contrast, neither peak CASE score nor antibody status showed a significant association with poor prognosis (*p* > 0.05).

## 4 Discussion

In this retrospective study, we analyzed the clinical characteristics, neuroimaging findings, and treatment strategies in a cohort of adult patients with autoimmune cortical encephalitis. We further investigated distinctive features based on antibody status and identified independent predictors of poor outcomes. Three major findings emerged. First, MOG antibodies account for approximately one‐fifth of autoimmune cortical encephalitis cases. Second, compared with the MOG‐CCE group, patients with non‐MOG antibody‐positive or with antibody negativity exhibited more severe clinical manifestations, a higher frequency of normal CSF findings, and an increased risk of relapse. Third, a higher peak mRS score was identified as an independent predictor of poor prognosis. These findings characterize the distinct clinic‐radiological phenotypes associated with different antibody statuses, rather than establishing a direct causal relationship between specific antibodies and cortical pathogenesis. These findings deepen our understanding of autoimmune cortical encephalitis with regard to disease management and prognostic prediction, particularly for MOG‐CCE.

In the past decade, the identification of various neuronal autoimmune antibodies has significantly expanded the spectrum of autoimmune cortical encephalitis. MOG antibodies have been paying increasing attention to their role in autoimmune cortical encephalitis. Prior case reports have delineated MOG‐CCE In the study by Ogawa et al. [4], four adult patients with MOG‐CCE predominantly presented with epileptic seizures, often accompanied by headache and low‐grade fever. Brain MRI demonstrated unilateral cortical T2/FLAIR hyperintense lesions associated with cortical swelling. In a subsequent case series, Pace et al. [5] reported that some patients with MOG‐CCE exhibited not only seizures but also focal neurological deficits, such as unilateral motor weakness or language disturbance. Furthermore, Ikeda et al. [20] provided pathological evidence supporting that MOG‐CCE constitutes a distinct clinical phenotype within the MOGAD spectrum. Twenty‐two patients (18.3%) in our cohort met diagnostic criteria for encephalitis and tested positive for MOG antibodies, a finding reported previously [8].

However, there are divergent views regarding the pathogenic role of MOG antibodies in cortical encephalitis. Lymphocytic infiltration was reported on brain biopsy specimens from two patients with MOG cortical encephalitis without a demyelination process, which may suggest that MOG antibodies do not play a primary pathogenic role [21]. By contrast, Höftberger et al. [22] reported that the pathological features of MOGAD often include cortical demyelinating lesions, differing from typical multiple sclerosis pathology and supporting a potential pathogenic role for MOG antibodies. Recent international consensus emphasizes that MOG antibody pathogenicity should be evaluated only in the context of high‐titer MOG‐IgG, compatible MRI lesions, and exclusion of alternative diagnoses [23]. In our cohort, all patients met these criteria: they were MOG‐IgG positive with median titers of 1:100 (Table S2), exhibited characteristic MRI cortical lesions including unilateral or bilateral T2‐FLAIR hyperintensities with swelling, and alternative causes were systematically excluded. Our study, aligned with prior reports, emphasizes that MOG antibodies are highly associated with this specific clinic‐radiological phenotype under rigorous diagnostic conditions [24]. Nonetheless, the primary objective of this study is to characterize antibody‐associated phenotypes rather than to establish a direct causal relationship between MOG‐IgG and cortical pathogenesis. Further pathological studies remain necessary to definitively confirm the pathological role of MOG antibodies in cortical encephalitis.

In our study, epileptic seizures were the most frequent presenting symptom in patients with MOG‐CCE (*n* = 11, 50%), consistent with previous reports identifying epilepsy as the predominant manifestation [4, 8]. However, in Christina's study [1], the number of patients whose initial symptom was headache (*n* = 15, 79%) exceeded those presenting with epilepsy (*n* = 13, 68%). This discrepancy may be attributable to the inclusion of pediatric patients in Christina's cohort, as children are more likely to seek medical attention for overt neurological symptoms such as headache [25]. Moreover, the incidence of psychiatric symptoms (36.7% vs. 22.7%) and disorders of consciousness (59.1% vs. 50%) was higher in the non‐MOG antibody‐positive group, with the antibody‐negative group showing a similar trend (57.1% vs. 22.7%). This distribution aligns with findings from other cohorts of acute disseminated encephalitis [24].

MRI‐based lesion distribution varies according to antibody status. The majority of patients with MOG‐CCE (68.2%) presented with unilateral lesions, consistent with findings reported by Wu et al. (75%) [26]. In 2019, Budhram et al. [8] described a rare phenotype of MOGAD characterized predominantly by epilepsy and cortical FLAIR hyperintensity involving one hemisphere, termed “FLAMES” (FLAIR‐hyperintense Lesions in Anti‐MOG‐associated Encephalitis with Seizures). However, Yao et al. [6] reported a predominance of bilateral involvement (80%, *n* = 8/10), a discrepancy that may be attributable to selection bias related to limited sample sizes. In our study, compared with the MOG‐CCE group, the non‐MOG antibody‐positive group predominantly exhibited unilateral lesions with frontal lobe involvement (55.1%), whereas the antibody‐negative group primarily demonstrated bilateral lesions with temporal lobe involvement (63.3%). These distribution patterns are consistent with previous reports [2] and correlate well with the predominant clinical manifestations observed in each group.

In our cohort, 45.8% of patients had poor functional outcomes (mRS ≥ 3 at the last follow‐up). Eight variables were considered potential risk factors for poor prognosis (mRS ≥ 3), including single clinical symptoms at disease onset, ICU admission, seizures, autonomic symptoms, psychiatric symptoms, impaired consciousness, peak CASE scores, peak mRS scores during the disease course, and autoimmune‐related antibodies positivity (compared with MOG antibodies). Previous studies have demonstrated that ICU admission, seizures, autonomic dysfunction, and mental or consciousness disorders are associated with adverse outcomes in univariate analyses. However, in a pediatric study by Kannan et al. [27], larger lesion size and longer treatment latency were associated with poorer prognosis. In Lee et al.'s study [12], diffuse cortical atrophy (DCA) or medial temporal lobe atrophy (mTA) was reported to be linked with adverse long‐term outcomes, whereas sustained immunotherapy appeared to improve prognosis in such patients. These differences may be attributable to variations in study populations and the variables analyzed. Peak mRS is the only independent predictor of poor outcome after multivariate analyses. Similarly, Balu et al. [28] reported that peak CASE or mRS scores serve as the strongest independent predictors of long‐term outcomes. These findings underscore that patients exhibiting more severe clinical symptoms during the peak period require heightened vigilance, as they may be at increased risk of poor prognosis and might benefit from more aggressive treatment.

In our cohort, the MOG‐CCE group exhibited the most favorable therapeutic response: only 22.7% experienced poor outcomes at the last follow‐up. The MOG‐CCE group also had the lowest CASE scores (1.5 [1–5] vs. 3 [1–5] vs. 2 [0–5]) and recurrence rate (22.7% vs. 40.82% vs. 61.2%) compared with the remaining two subgroups, consistent with prior reports demonstrating high glucocorticoid sensitivity in MOG‐CCE [26]. This differential response likely reflects distinct pathogenic mechanisms: neuronal autoantibodies (e.g., NMDAR, LGI‐1) primarily disrupt neuronal function through antibody internalization and immune complex formation, often resulting in irreversible injury [29], whereas MOG antibodies are B cell‐mediated, inducing demyelination and conduction block that is generally reversible following immunotherapy [30]. Acute phase treatment, like corticosteroids, effectively suppress B cell differentiation and antibody production but cannot readily remove antibodies or complexes that have entered neurons, which may account for the observed differences in therapeutic efficacy [31]. However, antibody status was not identified as an independent prognostic factor in subsequent multivariate analyses. This result may be attributed to two main factors. First, the initial titer was relatively modest without dynamic changes. Prio study reported that persistent or high titers of MOG‐IgG were associated with poor long‐term prognosis [32]. Second, the non‐MOG antibody‐positive group encompassed entities with vastly different clinical trajectories, ranging from the relatively favorable recovery often seen in anti‐LGI1 encephalitis to the poorer outcomes frequently associated with anti‐GABABR encephalitis [33, 34]. Such inherent intra‐group heterogeneity likely dilutes the subtype‐specific prognostic signals, making antibody status a less stable predictor than the peak mRS score.

AE associated with distinct neural autoantibodies is clinically heterogeneous, with differences in symptom profiles, MRI patterns, tumor associations, and CSF inflammatory changes [3]. For instance, anti‐NMDAR encephalitis often presents with psychiatric symptoms and seizures, and up to 50%–80% of patients have normal or nonspecific MRI findings, limiting the diagnostic value of MRI in this subtype [35, 36]. In contrast, GABABR [37] and AMPAR [38] encephalitis more commonly manifest as limbic encephalitis rather than primary focal cortical inflammation and may show more evident CSF pleocytosis, whereas LGI‐1 and CASPR2‐associated AE typically feature focal seizures and cognitive impairment with relatively modest CSF inflammation [39]. Accordingly, focal cortical encephalitis is not the canonical phenotype for each antibody subtype [3, 35, 36, 37, 38, 39], and cortical involvement may reflect atypical presentations, seizure‐related changes, overlapping immune‐mediated processes, or concomitant pathology.

Importantly, our study did not aim to characterize the full clinical spectrum of each antibody‐defined syndrome. Rather, by requiring cortical MRI abnormalities as an inclusion criterion, we deliberately selected an MRI‐defined cortical encephalitis phenotype within the broader autoimmune encephalitis spectrum. Therefore, the non‐MOG antibody‐positive group in our cohort represents an imaging‐selected subset with cortical involvement rather than the entirety of each antibody‐associated disease entity. Although this group is immunologically heterogeneous, the non‐MOG antibody‐positive group shared convergent clinical–radiological features, supporting a phenotype‐based comparison in the context of cortical encephalitis. However, several precautions are warranted when interpreting these pooled results. While this study approach underlined the statistical power to contrast MOG‐CCE against a broad autoimmune background, it may obscure subtle, antibody‐specific nuances. These results should be viewed as a collective autoimmune profile rather than implying biological homogeneity across all non‐MOG antibodies. To illustrate this diversity, representative cases of individual antibody subtypes within our cohort are detailed in Table S3.

Several limitations of this study warrant consideration. First, the retrospective, single‐center design may introduce selection bias and limit the generalizability of our findings. Second, the modest MOG‐CCE subgroup size limits statistical power of subgroup analyses, potentially obscuring true differences between groups and narrowing the captured clinical spectrum of this rare phenotype. Findings should therefore be interpreted as preliminary, pending validation in larger multicenter cohorts. Third, longer follow‐up periods are needed to adequately assess late relapses and the evolution of antibody status over time. Fourth, by pooling diverse non‐MOG antibody‐positive subtypes into a single comparison group, substantial clinical heterogeneity is introduced (Table S3), which may obscure antibody‐specific nuances and affect interpretation of pooled results. Larger cohorts are needed to statistically evaluate phenotypic differences among specific antibody subtypes. Fifth, the inclusion of antibody‐negative subgroup presents an inherent analytical challenge given its etiologic heterogeneity, as some cases may reflect autoimmune processes mediated by yet‐to‐be‐identified autoantibodies. To address this in future studies, we plan to implement tissue‐based autoantibody screening (TBA) on rat brain sections and expand our diagnostic panel to include MOG‐IgA and MOG‐IgM testing, potentially improving diagnostic resolution for currently seronegative cases. Finally, future multicenter, prospective studies with larger cohorts and extended follow‐up durations are warranted to more robustly investigate factors influencing clinical outcomes, disease progression, and the prognostic influence of antibody status.

Our findings carry important clinical implications for the early recognition and management of MOG‐CCE. When autoimmune cortical encephalitis is suspected, MOG antibody testing in both serum and CSF should be routinely included in the diagnostic workup. Given the favorable response to the immunotherapy of MOG‐CCE, prompt initiation of acute phase treatment should not be deferred pending antibody confirmation. For patients with severe acute presentations, long‐term maintenance immunotherapy with dynamic MOG‐IgG detection is advisable given the potential for unfavorable long‐term disability.

## 5 Conclusions

This study systematically analyzed the clinical, radiological, and prognostic characteristics of MOG‐CCE in adult patients. We demonstrated that MOG‐CCE represents a distinct phenotype within the MOGAD spectrum, characterized by seizure‐dominant presentation and unilateral cortical lesions on MRI. Patients with MOG‐CCE exhibited favorable responses to immunotherapy and generally achieved good long‐term functional outcomes. Compared with the non‐MOG antibody‐positive group, MOG‐CCE showed lower recurrence rates and milder residual disability. Notably, a higher peak mRS score during the acute phase was identified as the strongest independent predictor of poor prognosis, underscoring the importance of early recognition and timely immunotherapeutic intervention. These findings highlight the clinical significance of MOG‐IgG detection in identifying cortical phenotypes within the MOGAD spectrum and its value in guiding treatment decisions and prognosis. Future multicenter, prospective studies integrating MRI analysis and CSF biomarkers are warranted to clarify disease mechanisms and optimize individualized management strategies for MOG‐CCE.

## Author Contributions

Q.Y., B.H., K.S., W.D., J.W., L.Y., Y.D., and D.W. contributed to the conception and design of the study; all authors contributed to the acquisition and analysis of data; the final draft was revised and reviewed by Y.D. and D.W. All authors commented on previous versions of the manuscript. All authors read and approved the final manuscript.

## Funding

This work was supported by the Hunan Provincial Key Research and Development Program (grant no. 2021SK2027).

## Ethics Statement

This study has been approved by institutional review boards (IRBs) of the Second Xiangya Hospital of Central South University (2025‐ Z0014‐01). All procedures were conducted according to the guidelines of the Declaration of Helsinki and consents from participants or their legal guardians were obtained.

## Conflicts of Interest

The authors declare no conflicts of interest.

## Supporting information


**Table S1:** Comprehensive diagnostic work‐up performed to exclude alternative causes of cortical encephalitis.
**Table S2:** Distribution of antibody positivity by antibody type and specimen source.
**Table S3:** Clinical‐radiological and cerebrospinal fluid features of patients with different autoantibody profiles.

## Data Availability

Data generated or analyzed during the study are available from the corresponding author by request.
