# Supplementary material for: Myelin Oligodendrocyte Glycoprotein Antibody‐Associated Cerebral Cortical Encephalitis: A Comparative Study With Antibody‐Negative and Non‐MOG Antibody‐Positive Cortical Encephalitis in Chinese Adults
Source: CNS Neurosci Ther. 2026 May 6;32(5):e70915. doi: 10.1002/cns.70915 (PMC13148144; doi:10.1002/cns.70915)
Supplement: Supplementary file 1 — Table S1: Comprehensive diagnostic work‐up performed to exclude alternative causes of cortical encephalitis. Table S2: Distribution of antibody positivity by antibody type and specimen source. Table S3: Clinical‐radiological and cerebrospinal fluid features of patients with different autoantibody profiles. [file CNS-32-e70915-s001.docx]

| Examination | Diagnosis | Examination number | Completion rate | | Positive/abnormal rate | |  |
| --- | --- | --- | --- | --- | --- | --- | --- |
| **Infectious workup** | | | | | | |  |
| CSF PCR (HSV, VZV, HHV-6) | Viral encephalitis | 120 | 100% | | 0% | |  |
| CSF mNGS | CNS infection (viral, bacterial, fungal, parasitic) | 120 | 100% | | 0% | |  |
| CSF bacterial culture / Gram stain | Bacterial meningitis / meningoencephalitis | 120 | 100% | | 0% | |  |
| CSF fungal culture / India ink stain | Fungal or cryptococcal meningitis | 120 | 100% | | 0% | |  |
| CSF acid-fast bacilli stain | Tuberculous meningitis | 120 | 100% | | 0% | |  |
| **Metabolic/toxic workup** | | | | | | |  |
| Serum thiamine (vitamin B1) | Wernicke encephalopathy | 120 | 100% | | 0% | |  |
| Serum vitamin B12 | Metabolic encephalopathy / subacute combined degeneration | 120 | 100% | | 0% | |  |
| Thyroid function tests ± thyroid antibodies | Thyroid-related encephalopathy (SREAT) | 120 | 100% | | 0% | |  |
| **Prion disease workup** | | | | | | | |
| CSF 14-3-3 protein, tau, or RT-QuIC | Rapidly progressive dementia, including prion disease | 120 | 100% | | 0% | |  |
| **Paraneoplastic workup** | | | | | | | |
| Paraneoplastic antibodies (Hu, Yo, Ri, Ma2, CV2/CRMP5) | Paraneoplastic encephalitis syndromes | 120 | | 100% | 0% |  |  |

**Supplementary Table 1.** Comprehensive diagnostic work-up performed to exclude alternative causes of cortical encephalitis

**Supplementary Table 2.** Distribution of antibody positivity by antibody type and specimen source

| **Antibody status** | Threshold | | Median antibody titer | Range |
| --- | --- | --- | --- | --- |
| NMDAR | **CSF** | **≥1:1** | 1:100 | 1:3.2——1:1000 |
|  | Serum | ≥1:10 | 1:32 | 1:10——1:1000 |
| GABABR | **CSF** | ≥1:1 | 1:10 | 1:1——1:100 |
|  | Serum | ≥1:10 | 1:32 | 1:10——1:1000 |
| CASPR2 | **CSF** | qualitative only | 1:10 | 1:1——1:32 |
|  | Serum | ≥1:32 | 1:32 | 1:32——1:100 |
| AMPAR | **CSF** | ≥1:1 | 1:32 | 1:32 |
|  | Serum | ≥1:10 | 1:100 | 1:100 |
| LGI‐1 | **CSF** | **≥1:32** | 1:32 | 1:32 |
|  | Serum | qualitative only | 1:32 | 1:32 |
| mGluR5 | **CSF** | qualitative only | 1:10 | 1:10 |
|  | Serum | qualitative only | 1:10 | 1:10 |
| MOG | **CSF** | qualitative only | 1:32 | Negative——1:00 |
|  | Serum | ≥1:10 | 1:100 | Negative——1:1000 |

**Supplementary Table 3.** Clinical-radiological and cerebrospinal fluid features of patients with different autoantibody profiles

| Patients | | 1 | 2 | 3 | 4 | 5 | 6 |
| --- | --- | --- | --- | --- | --- | --- | --- |
| Gender | | M | F | F | 16 | 21 | 21 |
| Age (year) | | 35 | 67 | 43 | F | M | F |
| ICU | | - | + | - | - | - | - |
| Clinical manifestation | | Seizure; Psychiatric symptom;  Impaired consciousness; | Seizure;  Memory disturbance;  Psychiatric symptom;  Impaired consciousness;  Speech dysfunction | Seizure; Memory disturbance; Psychiatric symptom; Impaired consciousness; Speech dysfunction; Dyskinesia/dystonia | Seizure; Psychiatric symptom; Impaired consciousness; Dyskinesia/dystonia;Gait instability/ataxia | Seizure; Psychiatric symptom | Seizure; Psychiatric symptom; |
| Location of lesions in MRI | | Occipital lobe; Unilateral cortical | Temporal lobe; Frontal lobe; Unilateral cortical | Temporal lobe; Bilateral cortical | Frontal lobe;Unilateral cortical | Temporal lobe; Unilateral cortical | Parietal lobe; Unilateral cortical |
| CSF findings | Pressure  (mmH2O) | 150 | 140 | 160 | 180 | 170 | 98 |
|  | WBC count  (×10^6/L) | 60 | 26 | 3 | 0 | 2 | 3 |
|  | Protein (g/L) | 0.81 | 0.46 | 0.32 | 0.32 | 0.51 | 0.38 |
| Antibody status | | AMPAR | GABABR | NMDAR | CASPR2 | LGI‐1 | mGluR5 |
| Antibody titer  (serum) | | 1:100 | 1:100 | 1:100 | 1:32 | 1:32 | 1:10 |
|  | | 1:32 | 1:100 | 1:100 | 1:32 | 1:32 | 1:10 |
